# Activation of the Cholinergic Anti-Inflammatory Pathway as a Novel Therapeutic Strategy for COVID-19

**DOI:** 10.3389/fimmu.2020.595342

**Published:** 2021-02-08

**Authors:** Zhen Qin, Kefa Xiang, Ding-Feng Su, Yang Sun, Xia Liu

**Affiliations:** ^1^ Department of Clinical Pharmacy, School of Pharmacy, Second Military Medical University, Shanghai, China; ^2^ Key Laboratory of Molecular Pharmacology and Drug Evaluation (Yantai University), Ministry of Education, Yantai University, Yantai, China

**Keywords:** coronavirus disease 2019, cytokine storm, cholinergic anti-inflammatory pathway, therapeutic strategy, vagus nerve stimulation

## Abstract

The outbreak of coronavirus disease 2019 (COVID-19) underlined the urgent need for alleviating cytokine storm. We propose here that activating the cholinergic anti-inflammatory pathway (CAP) is a potential therapeutic strategy. However, there is currently no approved drugs targeting the regulatory pathway. It is evident that nicotine, anisodamine and some herb medicine, activate the CAP and exert anti-inflammation action *in vitro* and *in vivo*. As the vagus nerve affects both inflammation and specific immune response, we propose that vagus nerve stimulation by invasive or non-invasive devices and acupuncture at ST36, PC6, or GV20, are also feasible approaches to activate the CAP and control COVID-19. It is worth to investigate the efficacy and safety of the strategy in patients with COVID-19.

**Graphical Abstract:**
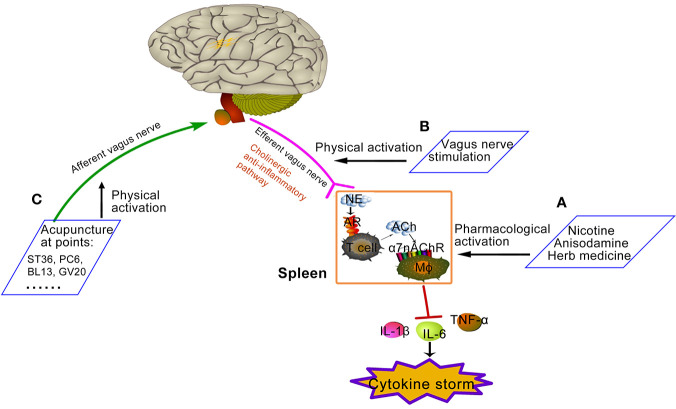
Proposed approaches of activating cholinergic anti-inflammatory pathway. **(A)** Pharmacological activation: Nicotine, anisodamine, and some Chinese herbs could direct or indirectly exert pharmacological activation of α7nAChR. **(B)** Physical activation: vagus nerve stimulation, such as invasive or non-invasive VNS device shown in **Figure 1**, increases the activity of cholinergic anti-inflammatory pathway. **(C)** Physical activation: some acupuncture points increase the activity of cholinergic anti-inflammatory pathway by activating the afferent vagal. Zu San Li (ST36) is located four finger widths down from the bottom of knee cap, along the outer boundary of shin bone. Nei Guan (PC6) is located three finger breadths below the wrist on the inner forearm in between the two tendons. Feishu (BL13) is on the back, 5 cm lateral to the lower border of the spinous process of the 3rd thoracic vertebra. Baihui (GV20) is on the center of the top of the head where the line connecting the high points of ears crosses the body midline.

## Introduction

A novel human coronavirus, severe acute respiratory syndrome coronavirus 2 (SARS-CoV-2), has been rapidly spreading across the world. SARS-CoV-2 is a single-strand, positive-sense, RNA virus and shares ~79% similarity of the genome sequence with human severe acute respiratory syndrome coronavirus (SARS-CoV) (1, 2). WHO has declared this coronavirus disease 2019 (COVID-19) a pandemic on 11 March, 2020. By Nov. 26, 2020, there have been 60,074,174 confirmed cases of COVID-19 globally, including 1,416,292 deaths (3). Most people infected with the SARS-CoV-2 will experience mild to moderate respiratory illness such as fever, cough, vomiting, diarrhea, and other symptoms, which do not need special treatment. A small part of patients, especially those with underlying medical problems such as obesity, diabetes, cardiovascular diseases, chronic respiratory diseases and cancer, are more likely to develop serious pneumonia, acute respiratory distress syndrome (ARDS), or multiple organ failure, resulting in considerable morbidity and mortality (4–6).

To date, no drugs have proven effective in reducing mortality, although some showed benefits in improving symptoms and shortening course. The Food and Drug Administration (FDA) has issued an emergency-use authorization (EUA) of remdesivir as the first drug approved to treat COVID-19 on Oct. 22, 2020 (7). However, due to lacking of evidence in the improvement of survival and other outcomes, WHO recommends against the use of remdesivir in COVID-19 patients (8). At present, there is also not yet an authorized vaccine to prevent COVID-19 in the United States (Updated Nov. 20, 2020) (9). Finding new effective COVID-19 treatments is still critical.

## Cytokine Storm: Lethal Factor and Therapeutic Dilemma

Accumulating evidence showed clinical and laboratory features of a cytokine storm syndrome in patients with severe COVID-19 (10). Cytokine storms are a common complication of infectious diseases, not only in COVID-19 but also in flu, SARS and MERS. Essentially, it is the overreaction of immune system to infection. For SARS-CoV-2, it enters the lungs, and its S protein specifically recognizes the host angiotensin-converting enzyme 2 (ACE2) receptor in alveolar epithelial type 2 cells. Upon binding, host serine protease TMPRSS2 cleaves the S protein and results in the fusion of the viral and cellular membranes, then SARS-CoV-2 enters into host cells (11). Consequently, the host initiates immune response and local inflammation to remove the virus. This viral infection–mediated local inflammatory response results in direct injury to the lung tissue, which is one of the proposed mechanisms behind the pulmonary manifestations of COVID-19 (11). However, in some patients, the release of cytokines is excessive or uncontrolled, leading to multiple organ failure, and a lethal consequence to the hosts (12). Before the effective vaccines or anti-viral drugs for COVID-19 are developed, anti-inflammation treatments are the urgent needs to calm cytokine storms and save lives (13, 14).

The existing, approved drugs with inflammation suppression are proposed as therapeutic options. Corticosteroids are getting special attention. However, their effects are still debatable, because they not only suppress lung inflammation but also impair the immune system’s ability to fight viruses. Clinical data did not support survival benefit from corticosteroids use in patients with coronavirus infection including SARS-CoV-2, even there are several possible harms (including delayed viral clearance) (15). Several anti-cancer drugs might be beneficial in states of overwhelming immune response like septic shock. Once again, due to immunosuppression, the present evidence is not enough to give any recommendations for routine clinical use (16). The effect of NSAIDs in COVID-19 patients is unknown (17). It can speculate that NSAIDs may have a beneficial effect in relieving symptoms caused by prostaglandins, but they do not directly affect the production of pro-inflammatory cytokines. A multi-source analysis in 2019 conducted by the CRPVs showed that NSAIDs administration increased the risk of bacterial infections (in particular pulmonary infections). At present, symptomatic treatment with NSAIDs is not recommended in patients with suspected COVID-19 cases (18–20). Some inhibitors or antibodies, based on their anti-inflammatory properties, are the potential agents for COVID-19 infection (e.g., JAK1/2 inhibitor baricitinib, IL-6 receptor blockade tocilizumab). Previous studies did not show a positive effect of these treatments in patients with cytokine storm of sepsis (21). Recently, John H. Stone et al. also reported that tocilizumab was not effective for preventing intubation or death in moderately ill hospitalized patients with COVID-19, suggesting that blocking single pro-inflammatory factor might not be sufficient to control cytokine storm (22). At present, the clinical trials of anti-IL-6 or anti-TNF therapy for COVID-19 are under the stage of being recommended or approved (23–25).

## Activating Cholinergic Anti-Inflammatory Pathway (CAP): A Novel Anti-Inflammation Strategy for COVID-19 Infection

Due to therapeutic dilemma of current drugs, more anti-inflammation approaches are needed. The CAP represents a neural mechanism of inflammation inhibition, first identified by Tracey KJ in 2000 (26). They found parasympathetic nervous system activity influences circulating TNF amounts and the shock response to endotoxaemia, which they call the ‘cholinergic anti-inflammatory pathway’ (26). The finding of CAP attracts considerable attention during the past 20 years and are well clarified now. In the presence of peripheral inflammation, afferent signals of vagal nerve are fired, notify the CNS and in turn activate an opposing efferent vagal nerve. The efferent vagus nerve then activates the splenic nerve to release its neurotransmitters including norepinephrine in the spleen. Subsequently, norepinephrine activates choline acetyltransferase-expressing T cells possibly *via* adrenergic receptors (AR), and promotes the production and release of T cell-derived acetylcholine (ACh). The ACh then interacts with α7 subunit-containing nicotinic receptor (α7nAChR) on macrophages and other immune cells, inhibits the release of pro-inflammatory cytokines and protects the body against damage. The efferent arm of this ‘inflammatory reflex’ is the CAP (27–29).

**Figure 1:**
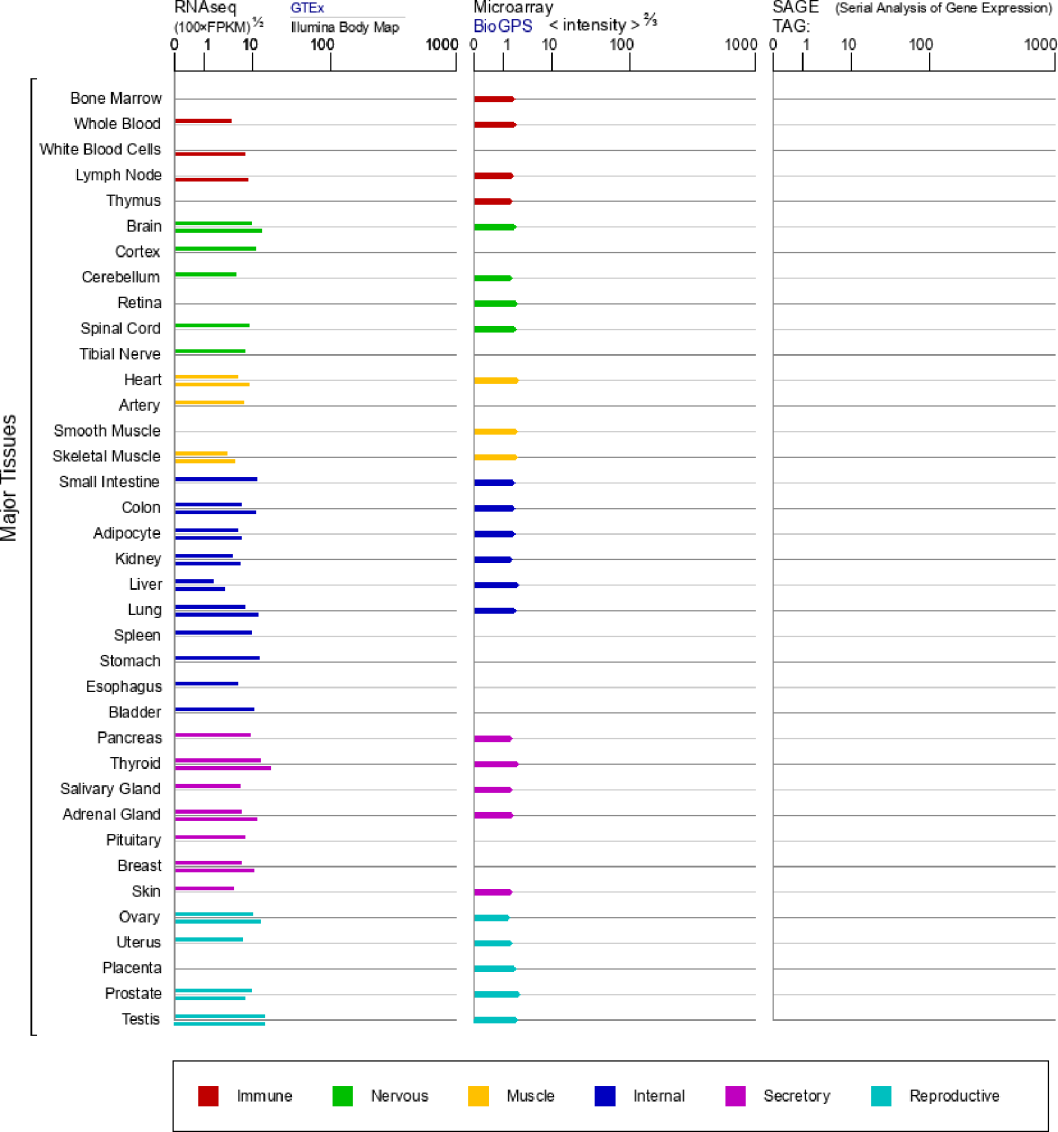
mRNA expression in normal human tissues from GTEx, Illumina, BioGPS, and SAGE for CHRNA7 Gene (https://www.genecards.org/cgi-bin/carddisp.pl?gene=CHRNA7&keywords=7nAChR).

The integrity of the inflammatory reflex is critically dependent on expression of the α7nAChR (27, 30). In addition to immune cells, α7nAChR is also wide-spread expressed in other different cells (**Figure 1**), including neurons and glial cells (31–33). In endotoxemia, the stimulation of vagus nerve attenuated systemic TNF levels in animals with α7nAChR deficiency in the nervous system, but failed in animals with an α7nAChR deficient immune system (30), identifying the α7nAChR expressed on macrophages and other immune cells as a main mediator of CAP output (28). Intracellular mechanisms are mainly involved in the suppression of NF-κB nuclear translocation, activation of a JAK2/STAT3 cascade, and inhibition of inflammasome activation triggered by the activation of α7nAChR on mitochondria, resulting in the inhibition of TNF, IL-1β, and other proinflammatory cytokines (34–37).

The regulation manner that neural inhibition in inflammation is faster, more effective and localized when compared to humoral ones. More importantly, it can simultaneously inhibit multiple proinflammatory cytokines, such as TNF, IL-1β, TNF-α, etc. Stimulation of the vagus nerve or activation of α7nAChR has been effective in attenuating the production of the pro-inflammatory cytokines and improving the survival of animals in various inflammatory diseases, especially sepsis. Recently, activating the CAP has also been suggested a therapeutic strategy for respiratory diseases (38). Therefore, this pathway is likely to be a hopeful therapeutic intervention in COVID-19 infection.

## Recommended Approaches That Activate CAP in COVID-19 Infection

### Pharmacological Activation

#### Nicotine

To date, no drugs of α7nAChR agonist have been approved yet. Nicotine is a nonspecific α7nAChR agonist. Many studies including ours have shown the protective role of nicotine in inhibiting the production of inflammatory cytokines and improving survival in experimental sepsis through the CAP (39–41). Nicotine-releasing enemas, gums, and patches have been used to treat ulcerative colitis in clinical trials (42–45). Therefore, nicotine might serve as a feasible drug in COVID-19 infection by activating the neuro-immune regulatory pathway.

Interestingly, a study from China Medical Treatment Expert Group reported that, of the 1,085 COVID-19 patients, 85.4% were non-smokers, while only 12.6% were smokers, and 1.9% were former smokers (46). Another study from a major Paris hospital found that few who had contracted the virus were regular smokers compared to the general population. They suggested a substance, possibly nicotine in tobacco, may prevent those smokers from catching COVID-19. Although other studies showed that smoking is, or not, associated with the prevalence or severity of COVID-19 (47, 48), French researchers are planning to examine whether nicotine patches could help prevent or lessen the effects of coronavirus (49).

However, WHO recently urged researchers, scientists and the media to be cautious about amplifying unproven claims that tobacco or nicotine could reduce the risk of COVID-19 (49). We must realize there exists an essential difference in pathological process between smokers and non-smokers with COVID-19 infection. Long-term smoking has harmed respiratory system, brought a high risk of cardiovascular diseases, cancer, diabetes, etc. When people are under these existing smoking-related disease conditions, it is difficult to distinguish the true role of nicotine. Furthermore, nicotine is different from tobacoo which contains a lot of other harmful ingredients. The detrimental effect of nicotine in different organ systems are debatable. For example, the harmful effects of smoking on the kidney is rather abundant, but the data assessing the singular effects of nicotine on the kidney are sparse (50). Conversely, nicotine is reported to protect kidney from renal ischemia/reperfusion injury through the CAP (51). In most of the tobacco-related diseases, nicotine is not regarded as a direct cause (52). Therefore, considering the emergent need is to control cytokine storm and save lives, nicotine could at least be tried in non-smoker COVID-19 patients under the careful monitoring in the function of important organs.

#### Anisodamine

Anisodamine, an active ingredient of Chinese herbal extracts, is a natural atropine derivative that has been isolated, synthesized and characterized in China. Like atropine and scopolamine, it is generally considered as a non-specific muscarinic cholinergic antagonist. Since 1965, anisodamine has been widely used clinically in China for the improvement of blood flow in circulatory disorders such as septic shock and disseminated intravascular coagulation (DIC). It could sharply reduce the mortality rate of various sepsis in clinic, such as fulminant epidemic meningitis, toxic bacillary dysentery, lobar pneumonia, etc. (53, 54).

Many mechanisms for the anti-shock action of anisodamine were proposed. Ruan et al. found anti-shock effect of anisodamine might be related to its anti-inflammation action (55). It can counteract LPS-induced endothelial cell activation by inhibiting the NF-κB pathway, which is a key molecule regulating the synthesis of pro-inflammatory cytokines. Now, series of studies suggested that its anti-inflammatory effect is mediated by indirectly activating the CAP. Li Q et al. found that the anti-inflammation and anti-shock role of anisodamine was significantly attenuated in LPS-treated IL-10-/- mice when compared to wide type mice. Further studies found anisodamine increased the expression of spleen α7nAChR in IL-10+/+ mice, but lose this role in IL-10-/- mice, indicating that anisodamine acts through upregulating α7nAChR synergistically with endogenous IL-10 (56). Furthermore, Liu C et al. found the protective role of anisodamine in shock and inflammation was disappeared in vagotomized, α7nAChR-deficient, or α7nAChR antagonist-treated septic mice, suggesting the involvement of the CAP. They speculate that anisodamine might block muscarinic receptor and reroute more ACh to bind to α7nAChR, therefore increasing ACh-mediated activation of α7nAChR (57, 58).

As an approved drug and indirect agonist of α7nAChR, anisodamine might be a feasible treatment for cytokine storm in COVID-19 infection. In addition to controlling inflammation, it might also provide additional advantages in COVID-19 patients. 1) COVID-19 and sticky phlegm in the lung: It was found a lot of sticky phlegm in the bronchioli and alveoli in COVID-19 death autopsy in China, which might be one of important lethal cause (59). According to the textbook of Pharmacology for medical students in China, anisodamine could antagonize M acetylcholine receptor, which will relieve smooth muscle spasm, inhibit gland secretion, dilate the bronchia and finally improve the function of pulmonary ventilation (60). 2) COVID-19 and abnormal coagulation: Studies have revealed that 71.4% of non-survivors of COVID-19 matched the grade of over DIC (35), indicating the deaths appear to be related to DIC (61). Anisodamine is well known for its dramatic therapeutic effect on DIC, in which the mechanisms are involved in the anti-platelet-aggregating, microcirculation-facilitating, thromboxane-B2-inhibiting, malondialdehyde-inhibiting, and 6-keto-PGF1 alpha-sparing effects (62). 3) Anisodamine and acute respiratory distress syndrome (ARDS): ARDS is a main lethal cause of severe COVID-19 patients (63). Anisodamine has been widely used to treat clinical and experimental ARDS in China since 1980s, and showed manifest therapeutic effect (64, 65). In 2003, anisodamine had also been used to treat patients of severe acute respiratory syndrome (SARS) with hypoxemia, and significantly decreased the mortality of SARS patients (66). 4) Anisodamine and organs protection: Anisodamine had displayed wide protection in other important organs, which were also affected in the COVID-19 infection. For example, anisodamine had cardioprotective effect through the suppression of cardiomyocytes apoptosis (67), protected against ischemic stroke *via* the α7nAChR (68), ameliorated renal dysfunction by reducing oxidative stress, the inflammatory response and cell death (69). The α7nAChR is widely distributed in these organs. Whether all these protective effects are related to the α7nAChR and CAP, and the effect of anisodamine in multi-organs damaged by COVID-19, should be further explored.

As a muscarinic antagonist, the adverse effects of anisodamine include reducing salivation, lacrimation and sweat, diminishing gastrointestinal motility, mydriasis, and increasing heart rate, which is tolerable and will disappear within 1–3 h (70). The usual treatment dose in humans is 10 mg/kg, yet doses as high as 500 mg/kg/day did not produce any serious adverse effects (71).

#### Herb Medicine

Many herbs have proven effective in the treatment of inflammatory diseases. With the development of modern medicine, their action mechanisms have been gradually determined. Especially, accumulating evidence showed that CAP plays a critical role in the anti-inflammation effect of some herbs, which provides the theoretical basis of modern medicine for their usage in COVID-19 infection.

1) Huang-Lian-Jie-Du decoction (HLJDD) is a Chinese formulation, and composed of rhizoma coptidis, radix scutellariae, cortex phellodendri, and fructus gardenia (72). It has been used for the treatment of sepsis over 1,700 years (73). In fighting against COVID-19, it is widely accounted as an effective medicine in China. Recent studies showed that HLJDD could affect the levels of ACh and choline, suggesting its anti-inflammatory effects possibly through its regulation in CAP (74). 2) Berberine is well known for its anti-inflammatory activity (75). It is an isoquinoline alkaloid extracted from rhizoma coptidis, and recently identified as an acetylcholinesterase inhibitor (76). It could inhibit acetyl cholinesterase enzyme (AChE) activity, increase ACh level and α7nAChR expression, therefore modulate CAP, inhibit inflammatory responses, and finally improve cognitive function and insulin resistance (77, 78). 3) Andrograph is frequently used to treat respiratory inflammatory diseases (79). 3-dehydroandrographolide (3-DA) is a natural andrographolide product from andrographis herba. Studies showed that 3-DA could bind with α7nAchR and protect against LPS-induced acute lung injury in mice. Methyllycaconitine, a α7nAChR specific inhibitor, could abolish this protective role (80). 4) Jiao-Tai-Wan (JTW), composed of coptis chinensis and cinnamon, is a famous prescription recorded in Han Shi Yi Tong, has been used for centuries for the treatment of insomnia (81). Recent studies found it could decrease the activity of acetylcholinesterase (AChE), increase the activity of choline acetyltransferase (ChAT), elevate the content of ACh, therefore activating cholinergic pathway and improving cognitive function (82). Its role in inflammation and COVID-19 infection is worthy of being explored. 5) Daikenchuto (DKT) is a gastrointestinal prokinetic Japanese herbal medicine (83). It contains four medical herbs: zanthoxylum fruit, processed dried ginger, ginseng, and malt sugar. Mari Endo et al. found that DKT could ameliorate inflammation in postoperative ileus by promoting ACh release from the cholinergic nerves, and this effect was partially suppressed in α7nAChR knockout mice (84).

Qing-Fei-Pai-Du decoction (QFPDD) was the most widely used prescription in China’s campaign to contain COVID-19 (85). Clinical observations suggested that QFPDD could significantly improve the abnormal laboratory indexes and clinical symptoms of COVID-19 patients, reduce the adverse reactions, and improve the therapeutic effect (86). QFPDD consists of 21 herbs including Ephedrae Herba, Glycyrrhizae Radix et Rhizoma Praeprata cum Melle, Armeniacae Semen Amarum, Gypsum Fibrosum, Cinnamomi Ramulus, Alismatis Rhizoma, Polyporus, Atractylodis Macrocephalae Rhizoma, Poria, Bupleuri Radix, Scutellariae Radix, Pinelliae Rhizoma Praepratum cum Zingibere et Alumine, Zingiberis Rhizoma Recens, Asteris Radix et Rhizoma, Farfarae Flos, Belamcandae Rhizoma, Asari Radix et Rhizoma, Dioscoreae Rhizoma, Aurantii Fructus Immaturus, Citri Reticulatae Pericarpium, and Pogostemonis Herba (87). Some compounds identified in QFPDD have been reported to act on targets of cholinergic synapse pathway. For example, earlier studies suggested that ephedrine could inhibit cholinesterases AChE (88), regulate acetylcholine receptor nAChR (89) and choline transporter CHT (90). These results suggest that QFPDD may regulate CAP to exhibit anti-inflammatory effects.

### Physical Activation

#### Vagus Nerve Stimulation (VNS)

VNS may act as a physical approach to treat COVID-19 infection by activating intrinsic CAP (26, 91, 92). In fact, VNS has been widely demonstrated to be a potentially anti-inflammatory therapy in experimental inflammatory disorder models such as sepsis, inflammatory bowel diseases, postoperative ileus and rheumatoid arthritis (93–96).

**Figure 2:**
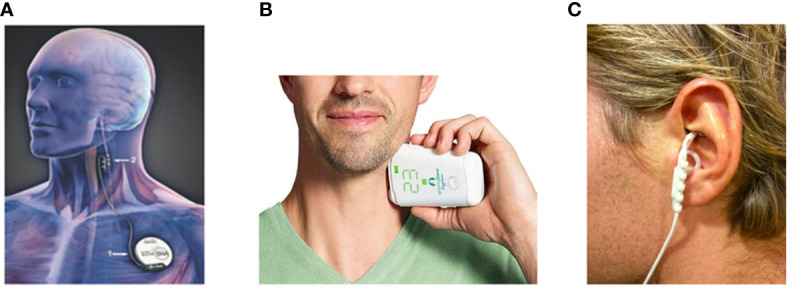
The device of vagus nerve stimulation. **(A)** The first device for vagus nerve stimulation (VNS), a pulse generator is placed in a subcutaneous pocket in the left chest wall and a spiral electrode wrapped around the left vagus nerve in the neck (97). **(B)** GammaCore, a handheld, self-contained non-invasive VNS (nVNS) device, directly contacts cervical skin surfaces and delivers electrical signal to the vagus (98). **(C)** NEMOS, an external device that provides transcutaneous VNS (tVNS) by using a dedicated intra-auricular electrode, stimulates the auricular branch of the vagus nerve (99).

VNS is already approved by FDA for the treatment of drug-resistant epilepsy and depression. The first device for VNS is invasive, which needs an electrode surgically implanted in the neck around the left vagus nerve (**Figure 2A**). Generally, high-frequency (20–30 Hz) stimulation is used to treat epilepsy and depression, whereas low-frequency (1–10 Hz) stimulation is used to activate the CAP in animal models (97). ClinicalTrials.gov, a service of the US National Institutes of Health (100), has received total 148 registrations of clinical studies on VNS. These studies are conducted in patients with epilepsy, depression, stroke, pain, diabetes, heart failure, and also inflammatory diseases, i.e. rheumatoid arthritis and inflammatory bowel diseases, demonstrating the interest for such a procedure in various health domains.

Such invasive VNS involves an additional intervention with surgical exposure of the vagus nerve. Most of the cases will encounter no problems. Perioperative complications of VNS are mostly related with cardiac dysrhythmias, which could be adjusted by elongating the off period (101). For severe COVID-19 patients, careful evaluation and monitoring in cardiovascular system are still necessary. The most frequently acute complications of VNS implantation include temporary excessive salivation, mild coughs, paralysis of the vocal cord, lower facial weakness, the coercive feeling of coughing, rarely bradycardia, and very rarely, asystole, all of which are reversible (102). The infection at the implantation site is a rare complication (103).

Non-invasive VNS devices (nVNS) has become the latest hot spot in recent years and are widely used for sick individuals with relative safety and tolerability, such as GammaCore and NEMOS (104). GammaCore (electroCore LLC, Basking Ridge, NJ, USA) is a handheld, self-contained nVNS device approved by FDA, which directly contacts cervical skin surfaces and delivers electrical signal to the vagus (**Figure 2B**). This device was under investigation for headache, epilepsy and gastrointestinal disorders (105). Recently, it was clinically used to treat respiratory symptoms associated with COVID-19 in two patients, and showed a clinical benefit (98). In August 2020, the GammaCore Sapphire CV (nVNS) received an Emergency Use Authorization (EUA) from the FDA to treat patients with known or suspected COVID-19 associated asthma and respiratory distress with decreased blood oxygen (106).

NEMOS (Cerbomed, Erlangen, Germany) is an external device that provides transcutaneous VNS (tVNS) by using a dedicated intra-auricular electrode (like an earphone) which stimulates the auricular branch of the vagus nerve (**Figure 2C**) (99). Studies showed that auricular VNS could suppress LPS-induced inflammatory responses *via* α7nAChR-mediated CAP (107), indicating an intimate connection between auricular concha and efferent vagus nerve. Hence, it is also worthy of being tried in COVID-19 patients.

#### Acupuncture

Acupuncture, an important part of traditional Chinese medicine (TCM), has been used in China for the treatment of a variety of conditions for at least 5,200 years. It is nowadays widely used throughout the world including Asia, Europe, and the USA. Acupuncture is one of the most popular complementary and alternative therapies, and has steadily claimed its usefulness (108). Therapeutic effects of acupuncture in inflammatory diseases have also been widely reported, with manual operation or electro-stimulation at varying acupuncture points (109).

The foundation of acupuncture is based on a complex meridians theory according to TCM. Modern physiologists have raised a “neural hypothesis” that acupuncture could primarily stimulate sensory nerves close to the inserting needle underneath the skin, transmit signals to brain, and produce clinical influence. This is the most rational basis for defining meridians (110). Some acupuncture points (such as ST36, PC6, and GV20) have been demonstrated to increase the vagal activity in experimental animals and human subjects (111–113). Furthermore, its systemic anti-inflammatory action has been highly proposed to be mediated by the CAP (114). Experimental data showed that acupuncture at Zusanli (ST36) and Feishu (BL13) points could reduce lung inflammation and improve lung function in COPD (chronic obstructive pulmonary disease) rats (115), at the acupoint “Baihui (GV20)” could attenuate cerebral inflammation and ischemic injury in MCAO (middle cerebral artery occlusion) rats (116). When α7nAchR was blocked, these effects were revered.

Auricular branch of vagus nerve is a special vagal branch that innervates the body surface (117). Considering transcutaneous auricular VNS is effective in inflammation suppression *via* the CAP (107), the application of auricular acupuncture by pressing pills (cowherb seed) on ear acupoint, which is widely used in practice, is also recommended for the COVID-19 infection.

## Conclusion

Accumulating evidence showed that the high level of cytokines indicates a poor prognosis in COVID-19, although there are some controversy supported by a few clinical studies (118). Here, we propose a potential therapeutic strategy to fight against cytokine storm in COVID-19 infection, namely activating organism’s intrinsic CAP. We also provide a systemic approach (Graphical abstract) including pharmacological activation (from modern medicine to herb medicine) and physical stimulation (from VNS to acupuncture), which are all feasible treatments against COVID-19 infection at this moment. Clinical studies are worthy of being carried out to investigate the efficacy and safety of the therapy in human subjects. Many of the pre-approved drugs and methods might be particularly valuable for use under emergency and for patients in developing and under-developed countries.

## Data Availability Statement

The original contributions presented in the study are included in the article/supplementary material. Further inquiries can be directed to the corresponding authors.

## Author Contributions

ZQ and KX collected the literature and drafted the paper. XL put forward the hypothesis and revised the manuscript. YS and D-FS participated in discussions. All the authors had final responsibility for the decision to publish. All authors contributed to the article and approved the submitted version.

## Funding

This work was supported by National Science and Technology Major Project, National Natural Science Foundation of China (2018ZX09711002-003-015, 81773726 to XL, and 82003981 to ZQ), National Key Research and Development Program of China (2020YFC0845400, 2017YFC1700200 to Weidong Zhang), Shanghai Sailing Program (19YF1459500 to ZQ) and Science and Technology Innovation Action Plan Project (19401900100 to XL and 19431901400 to ZQ).

## Conflict of Interest

The authors declare that the research was conducted in the absence of any commercial or financial relationships that could be construed as a potential conflict of interest.

## Abbreviations

ACh, acetycholine; AChE, acetyl cholinesterase enzyme; CAP, cholinergic anti-inflammatory pathway; ChAT, choline acetyltransferase; COVID-19, coronavirus disease 2019; DIC, disseminated intravascular coagulation; DKT, Daikenchuto; HLJDD, Huang-Lian-Jie-Du decoction; JTW, Jiao-Tai-Wan; QFPDD, Qing-Fei-Pai-Du decoction; SARS-CoV-2, severe acute respiratory syndrome coronavirus 2; TCM, traditional Chinese medicine; VNS, vagus nerve stimulation; α7nAChRs, α7 subunit-containing nicotinic receptors.
